# Impact of flexion versus extension of knee position on outcomes after total knee arthroplasty: a meta-analysis

**DOI:** 10.1007/s00402-016-2613-7

**Published:** 2016-12-27

**Authors:** Chao Jiang, Jieqiong Lou, Wenwei Qian, Canhua Ye, Shibai Zhu

**Affiliations:** 1 Department of Orthopaedic Surgery, Peking Union Medical College Hospital, Peking Union Medical College, Chinese Academy of Medical Science, 100730 Beijing, China; 2 School of Public Health, Shanghai Jiao Tong University, Shanghai, China

**Keywords:** Total knee arthroplasty, Postoperative knee position, Range of motion, Blood loss

## Abstract

**Introduction:**

Controversy still exists regarding positioning of the knee in flexion or in extension after total knee arthroplasty (TKA) impacts treatment outcomes. In this meta-analysis, we evaluated if a postoperative knee position regime could positively affect the rehabilitation.

**Methods:**

A comprehensive search for randomized controlled trials (RCTs) assessing the effect of knee positioning after TKA was conducted. The outcomes of interest were blood loss and range of motion (ROM); total calculated blood loss (CBL), drainage volume, hidden blood loss (HBL), decline of hemoglobin level and requirement for blood transfusion.

**Results:**

Ten RCTs involving 962 knees were eligible for meta-analysis. Positioning the knee in flexion after TKA was significantly associated with lesser CBL (*P* < 0.00001), less HBL (*P* < 0.00001) and decreased requirement for blood transfusion (*P* = 0.06). On subgroup analyses, the flexion group was found to have significantly less decrease in hemoglobin level 48 h to 6 days after surgery (*P* = 0.003), while no significant difference was noted at 24 h after surgery (*P* = 0.29). Further,a superior ROM was observed in flexion group (5–7 days after surgery) (*P* = 0.002), while there was no significant difference at 6 weeks. No significant inter-group difference in wound drainage was observed at 24 h after surgery.

**Conclusion:**

Positioning the knee in flexion in the early postoperative stage was associated with significantly lesser CBL, lesser HBL, decreased requirement for blood transfusion and better ROM at least in the early postoperative period, which may contribute to early rehabilitation. However, no significant difference was found in ROM at 6 weeks.

## Introduction

As one of the most successful orthopaedic procedure for treating most end-stage knee diseases, total knee arthroplasty (TKA) is associated with pain relief, restoration of function, and recovery of mobility [1]. Since most candidates for TKA are elderly patients, the co-morbid medical problems often lead to decreased tolerance to surgery. Postsurgical complications, including substantial blood loss are common after TKA. Optimal perioperative management has long been a major focus of medical practice and research for better blood management and rapid rehabilitation. The commonly used interventions include tranexamic acid (TXA) [2], use of tourniquet ing [3], cryotherapy [4], drainage protocols [5], cruciate-retaining prosthesis [6], patient-specific instrumentation (PSI) [7], minimally invasive approach, computer-aided surgical techniques [8], continuous passive motion (CPM) and postoperative knee position regimens [9].

Positioning the knee in flexion in the early postoperative stage (compared with extension) have been reported to be simple and cost-effective way to improve patient outcomes and satisfaction, whilst decreasing the strain on hospital resources, such as requirement for staff, beds, blood transfusion and expensive equipment [10]. However, recent studies about the effects of these two different knee position regimens (flexion versus extension) have reported conflicting results [11].We conducted a meta-analysis of RCTs only to assess the effect of knee position after TKA on patient outcomes, as an aid to inform optimal postoperative management strategy.

## Materials and methods

### Search strategies

The systematic review was conducted according to the Quality of Report of Meta-Analyses of Randomized Clinical Trials (QUOROM) recommendations [12]. A comprehensive search for studies about knee position was conducted through the online database of PubMed, ScienceDirect, Cochrane Library, CNKI, Wanfang Med Online and VIP, from inception till June, 2015. The following key words were used: (“total knee arthroplasty” or “total knee replacement”) in combination with “postoperative management”, “knee position”, “extension”, “flexion”. References from these publications were manually scrutinized for additional relevant articles. The duplicated articles were eliminated using Endnote software (EndNote X6).

### Study selection

All RCTs comparing knee positioning in flexion versus extension after primary TKA were eligible for inclusion. If there were more than one eligible publication from one trial, the one with higher quality data, or the one with most recent publication date, was included. The exclusion criteria were: non-randomized and quasi-randomized trials, trials involving bilateral TKA, or those performed within a 3-month interval, disordered hemostasis due to any cause, history of peripheral vascular disease, time of flexion >72 h and angle of flexion >90°.

### Outcomes of interest

The primary outcomes were blood loss and ROM. CBL, HBL, drainage volume, drop in HB level and requirement for blood transfusion were considered for assessment of blood loss.

### Assessment of methodology

Two reviewers (Jiang C and Lou JQ) independently conducted the quality assessment of study methodologies with respect to study design, methodology for identification of study groups, confirmation of subsequent data, and fairness of comparisons [13].

### Data extraction

Two reviewers independently retrieved clinical studies and extracted the data from eligible trials. Any disagreement was resolved by consensus with the senior review author, and reasons for exclusion documented. Any additional data required for the meta-analysis was obtained from the primary authors through e-mail communication.

### Statistical analysis

For each included study, the weighted mean differences (WMD) at 95% Confidence intervals (CI) were calculated for continuous outcomes, while odds ratio (OR) at 95% confidence intervals (CI) were calculated for dichotomous outcomes. Statistical heterogeneity was assessed using the *I* ^2^ value and Chi-square test. A *P* value >0.1 and an *I* ^2^ value ≤50% were considered indicative of a lack of statistical heterogeneity, and fixed-effects model was used to estimate the overall effect size. In others, random-effects model was adopted and a subgroup analysis or sensitivity analysis was performed. Two kinds of subgroup analyses (according to time) were performed. All analyses were performed using the Review Manager Software (Revman 5.3) and *P* value <0.05 was considered as statistically significant.

## Results

### Characteristics of selected studies

**Fig. 1:**
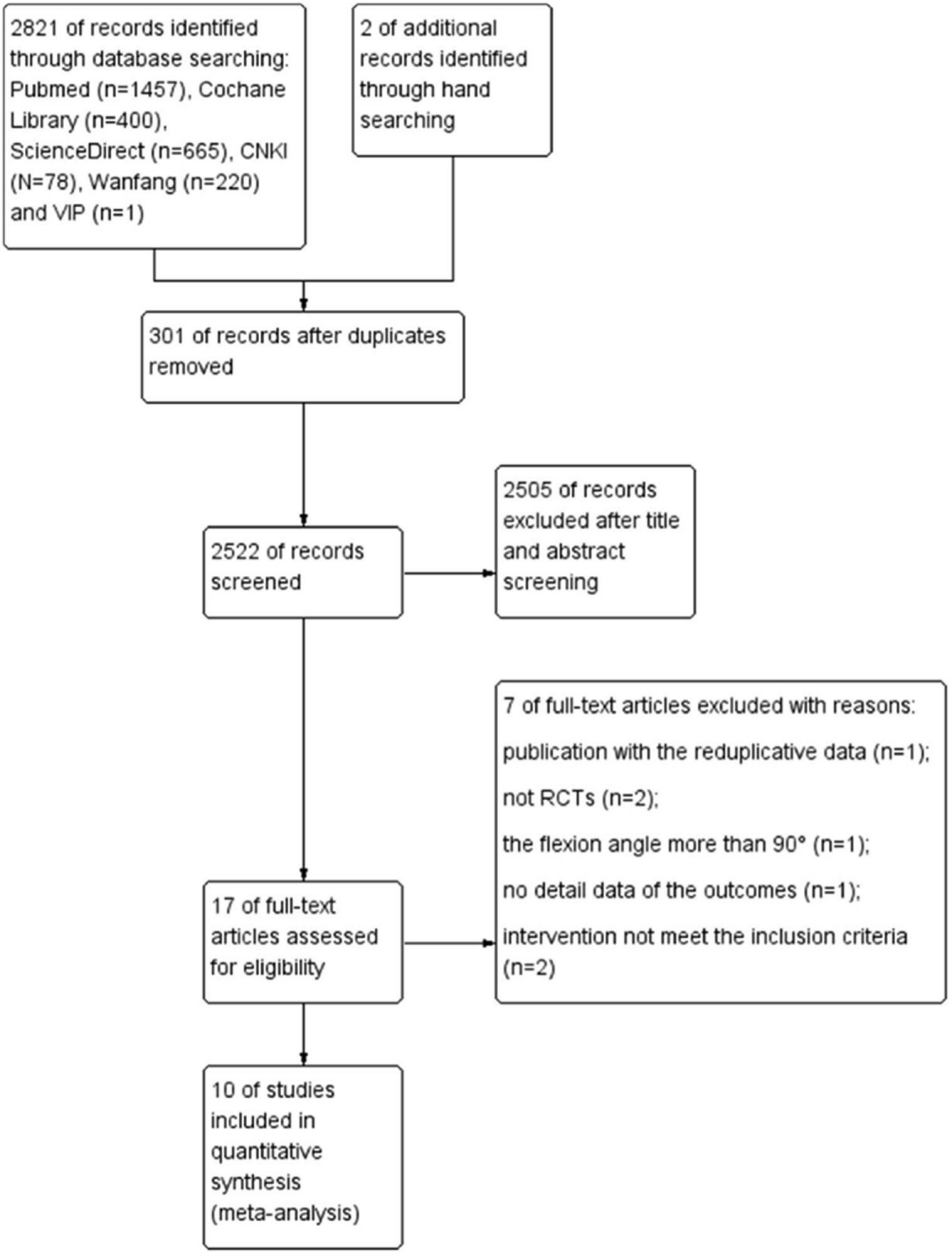
Schematic illustration of literature search and study selection

The details of the search and exclusion criteria are illustrated in Fig. 1. Ten RCTs [11, 14–22] published between 2003 and 2015, were finally selected. A total 962 knees ranging from 40 to 221 in each trial came within the purview of this meta-analysis. There were no significant inter-group differences with regard to age, gender and body mass index (BMI). The baseline level of hemoglobin [11, 14, 17–19, 21, 22] and ROM [11, 14, 20, 22] were well balanced between the flexion group and extension group in some studies. Three RCTs were excluded due to various reasons. These included duplication of data of one study [23] with another study [11]. From amongst these two studies, the one with better data quality and with the larger sample size was included. Speck et al. [9] were the first team who conducted RCT on this subject; however, the study was excluded due to lack of availability of complete data. RCT reported by Napier et al. [24] was excluded as the flexion angle was 120°, which was much more than that in the other RCTs included in the meta-analysis.

The characteristics of the studies included in the meta-analysis are summarized in Table 1, while the study design and the approach used in each of the RCT is shown in Table 2.

**Table 1:** Key characteristics of studies included in the meta-analysis:

Included studies	Study period	Knees enrolled (flexion/extension)	Knees analyzed (flexion/extension)	Male/female		Demographics
				Flexion	Extension	
Ong [16]	2000–00	20/20	20/20	8/12	7/13	No difference
Ma [14]	2005–06	50/50	49/46	24/25	24/22	No difference
Wang [19]	2008–09	30/30	30/30	NA	NA	No difference
Madarevic [15]	2008–09	18/56		NA	NA	No difference
Li [11]	2008–09	55/55	55/55	15/40	17/38	No difference
Hu [18]	2010–12	65/68	65/68	0/65	0/68	No difference
Panni [17]	2012–13	50/50	50/50	0/68	10/40	No difference
Zhao [20]	2011–13	119/102	119/102	0/119	0/102	No difference
Antinolfi [21]	NA	20/20	20/20	7/13	10/10	No difference
Liu [22]	2013–14	50/50	50/50	16/34	18/32	No difference
Total		477/501	474/488			

*NA* not available

**Table 2:** Study design and treatment protocol of studies included in the meta-analysis

Included studies	Type of study	Flexion			Approach	Patellar resurfacing	Type of prosthesis
		Knee	Hip	Time			
Ong [16]	RCT	70°	35°	6 h	NA	NA	Depuy LCS/PFC
Ma [14]	RCT	70°	70°	24 h	MP	NA	Smith&nephew GII PS
Wang [19]	RCT	45°	60°	24 h	MP	NA	Smith&nephew GIIPS
Madarevic [15]	RCT	90°	NA	6 h	MP	NA	NA^a^
Li [11]	RCT	30°	30°	72 h	MV	no	Smith&nephew GII PS
Hu [18]	RCT	70°	45°	12 h	MP	no	Stryker PS
Panni [17]	RCT	90°	45°	6 h	MP	NA	Zimmer Nexgen PS
Zhao [20]	RCT	45°	30°	24 h	MP	no	Stryker PS
Antinolfi [21]	RCT	90°–50°^b^	NA	3–3 h^b^	MP	no	Smith&nephew Journey
Liu [22]	RCT	45°	45°	NA	MP	No	Smith&nephew GII PS

*NA* not available, *MV* mid-vastus approach, *MP* medial parapatellar approach

^a^Not available, but the same prosthesis was used in the two groups

^b^Knee was bent to 90° for the first 3 h post-surgery, and the kept at 50° for the next 3 h

### Risk of bias

**Fig. 2:**
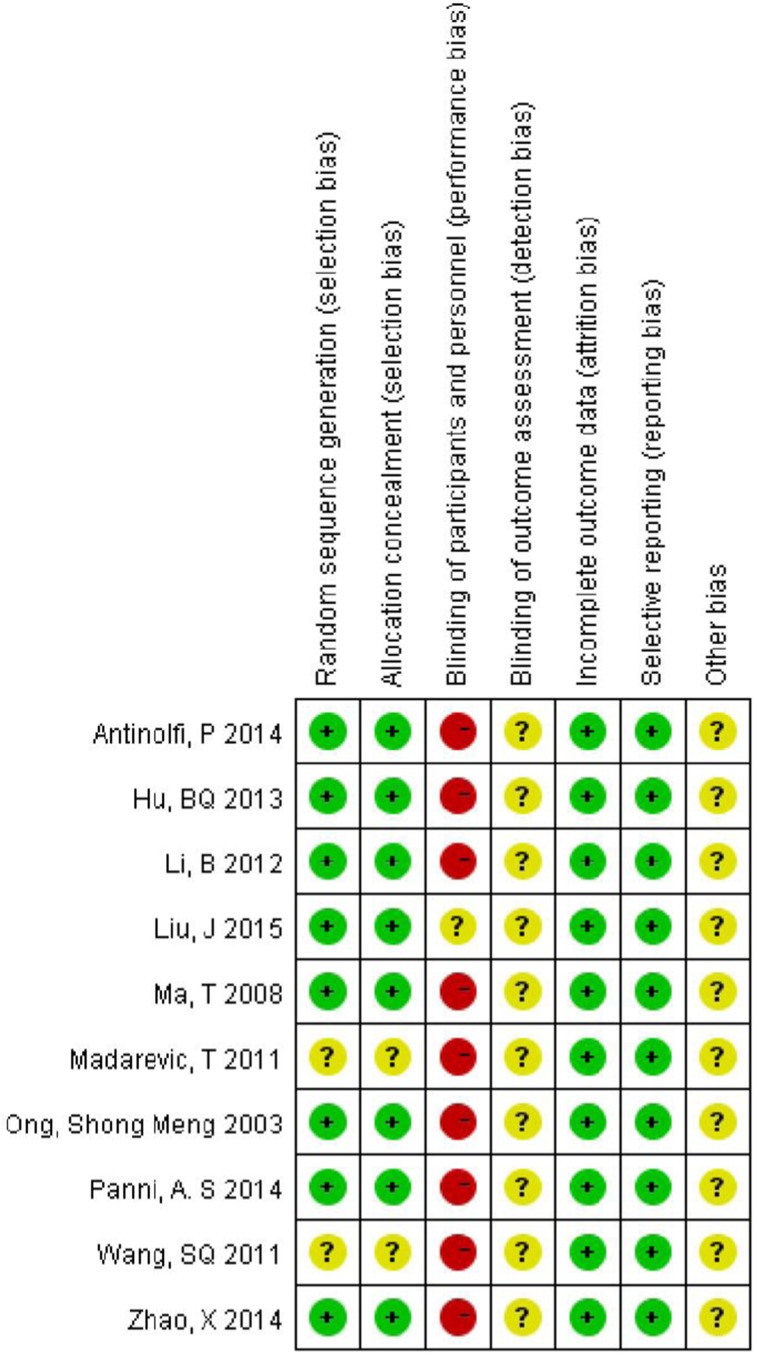
Quality assessment of risk of bias in included studies

In general, the methodological quality had moderate risk of bias as per the Cochrane Collaboration recommendations. Randomization was done using the random number list in four studies [11, 17–22]; the closing envelope technique [14, 16] and calendar randomization [15] were used in the remaining three. The detailed risk of bias in methodological quality of included studies is elaborated in Fig. 2.

### Meta-analysis results

#### Blood loss

All the drainage volumes were measured at 24 h after surgery; we chose calculated total blood loss (CBL), drainage volume and hidden blood loss (HBL) as measures for blood loss. The flexed position was associated with a significantly lower CBL (MD = −208.08 mL, 95% CI −299.80, −116.37, *P* < 0.00001) (Fig. 3.1), and HBL (MD = −132.15 mL, 95% CI −183.29, −81.00, *P* < 0.00001) (Fig. 3.3), as compared to that in the extended position. However, there was no significant difference between the flexion and extension groups with regard to drain volume at 24 h (MD = −53.32 mL, 95% CI −116.77, 10.13, *P* = 0.10) (Fig. 3.2). Although we could in general see the heterogeneities, the sensitivity analysis showed that there was no indication for subgroups.

**Fig. 3:**
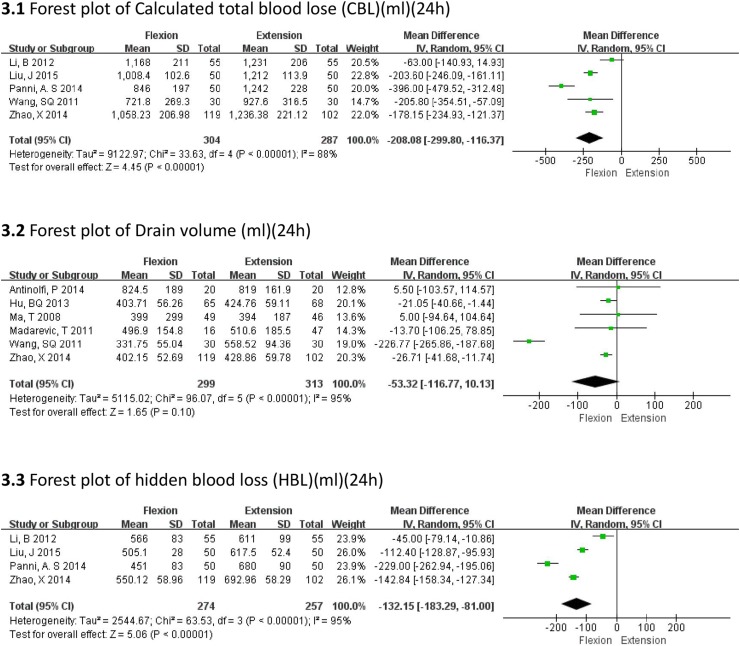
Forest plots of blood loss (mL) (24 h)

### Fall in hemoglobin

**Fig. 4:**
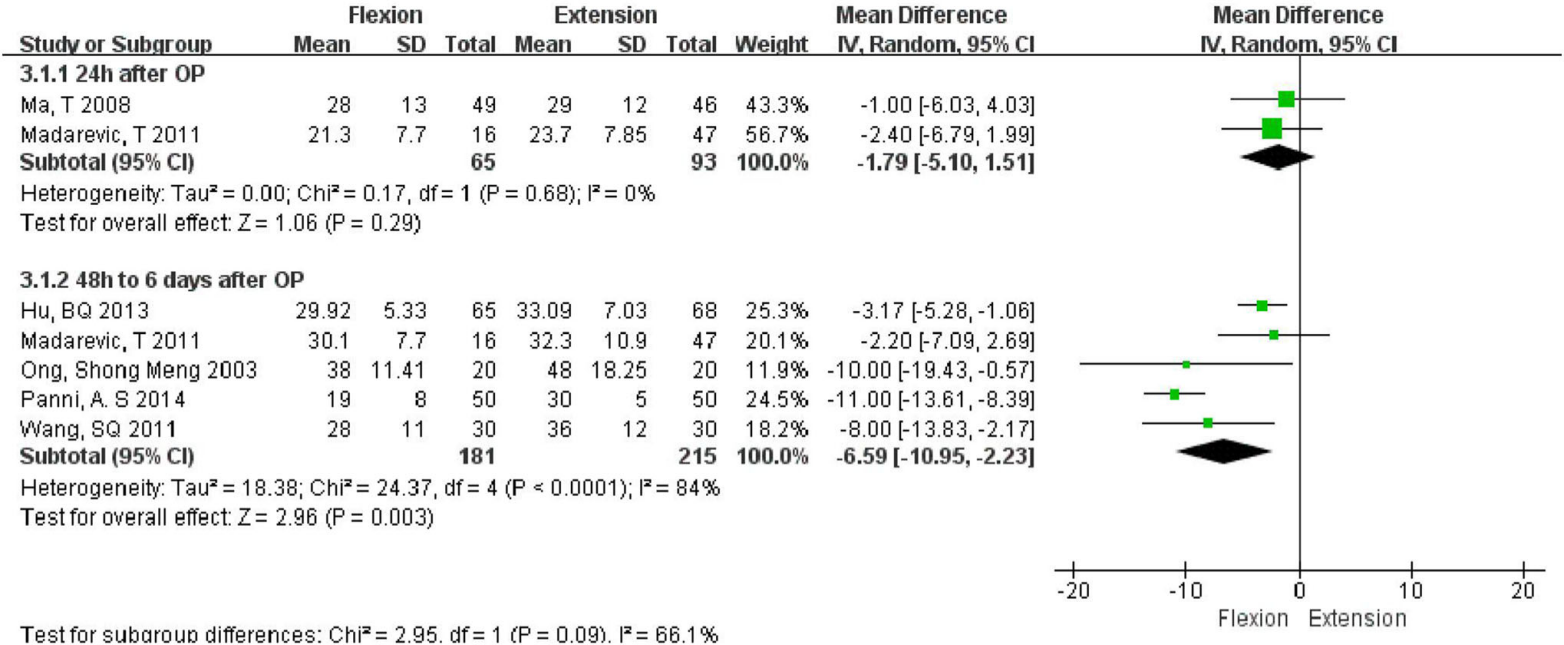
Forest plot of fall in hemoglobin (g/L)

As the fall in hemoglobin level was measured at different times, we performed the subgroup analysis to reduce the heterogeneity caused due to measurement at different times. On subgroup analyses, the flexion group was found to have a significantly less fall in hemoglobin as compared to that in the extension group (MD = −6.59 g/L, 95% CI −10.95, −2.23, *P* = 0.003) 48 h to 6 days after surgery, while no significant inter-group difference (MD = −1.79 g/L, 95% CI −5.10, 1.51, *P* = 0.29) was noted between the two groups at 24 h after operation (Fig. 4).

### Number of patients requiring transfusion

**Fig. 5:**
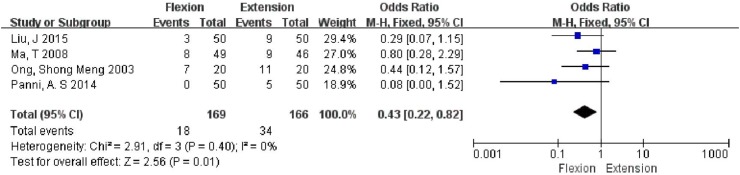
Forest plot showing number of patients requiring transfusion

As 4 studies involving 335 knees reported the results without significant heterogeneity (*P* = 0.40; *I* ^2^ = 0%), a fixed-effects model was used. A significantly lower number of patients required blood transfusion in the flexion group as compared to that in the extension group (OR 0.43, 95% CI 0.22, 0.82; *P* = 0.01) (Fig. 5).

### Range of motion (ROM)

**Fig. 6:**
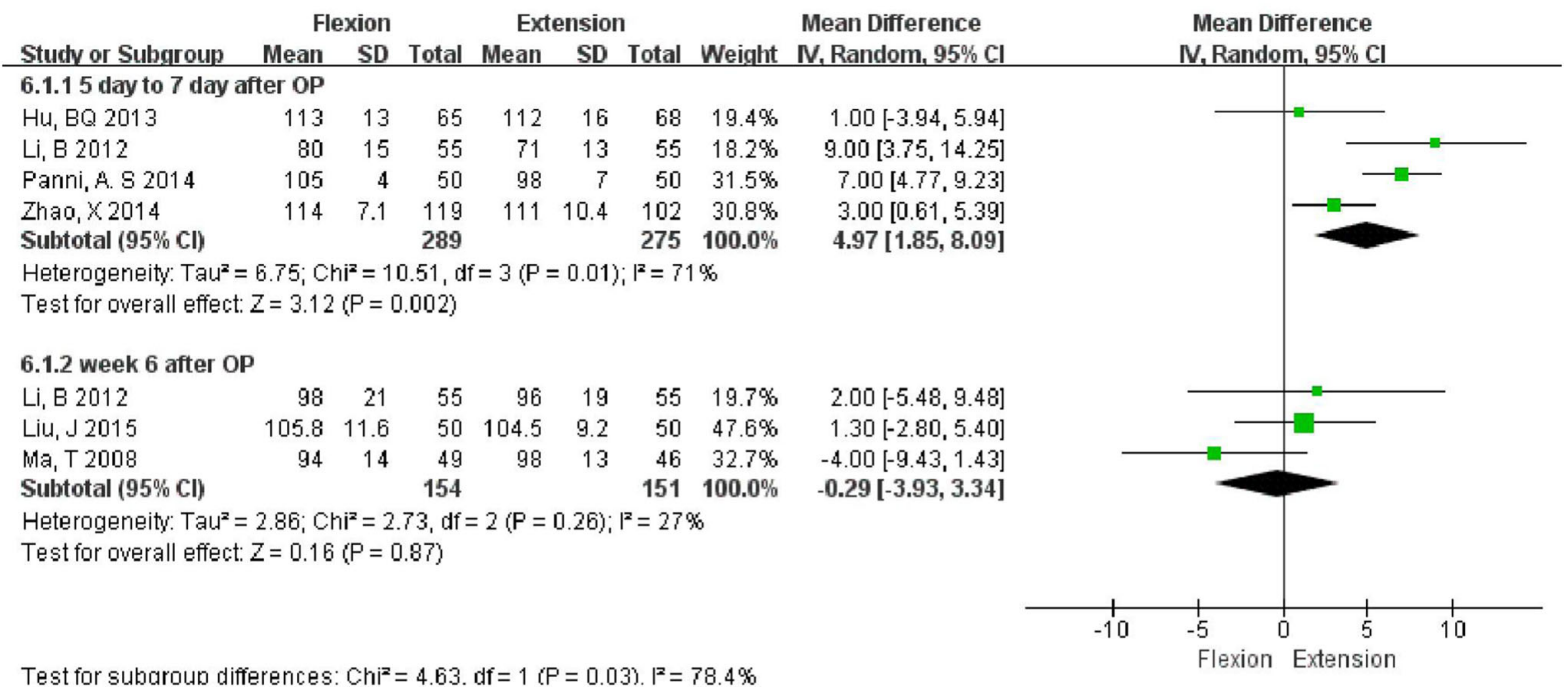
Forest plot of Range of motion (ROM)

Subgroup analysis was carried out at two time points (5–7 days and 6 weeks after surgery). The ROM was found to be significantly different in the flexion group (MD = 4.97°, 95% CI 1.85, 8.09, *P* = 0.002) at 5–7 days after surgery when compared to extension group. However, at 6 weeks after surgery, no significant difference (MD = −0.29°, 95% CI −3.93, 3.34, *P* = 0.87) in ROM was noted between the two groups (Fig. 6).

## Discussion

Substantial blood loss during TKA often requires allogeneic blood transfusion. However, the potential risk [25] and higher costs associated with allogeneic blood transfusion propel surgeons to find ways to reduce the blood loss during surgery. The first RCT on this topic was published by Speck et al. [9], which found that 70° of knee flexion for 6 h after the cemented TKA significantly reduced blood loss (by 30%) as determined by wound drainage. A few studies [9, 17] have proposed that the knee position after TKA is a simple and cost-effective way to maximize patient outcomes without increasing the side effects, however, the results of these studies are debatable. To clarify this issue, we performed the present meta-analysis, which included recent RCTs comparing postsurgical flexed knee position with extended knee position. It is possible that the different hip and knee angles in different trials might impact the results. When the angles of hip and knee are too large or over-extended, the resulting strain on the vein might hinder the blood backflow [11], so we only included studies in which the hip or knee angles were no more than 90°.

We chose the CBL, HBL, drainage volume, fall in hemoglobin, and requirement for blood transfusion as the parameters indicative of postoperative blood loss. The flexion group was found to have significantly less CBL (MD = −208.08 mL, 95% CI −299.80, −116.37, *P* < 0.00001), and HBL (MD = −132.15 mL, 95% CI −183.29, −81.00, *P* < 0.00001), as compared to that in the extension group. It appears that the flexion group had less hidden blood loss which is partially presents as limb swelling and ecchymosis [26]. Although no significant inter-group differences were observed in terms of fall in hemoglobin (MD = −1.79 g/L, 95% CI −5.10, 1.51, *P* = 0.29) at 24 h after operation, the flexion group appeared to be at an advantage from 48 h to 6 days after operation as the decline of hemoglobin was less in this group (MD = −6.59 g/L, 95% CI −10.95, −2.23, *P* = 0.003). One reason for this observation could be that only two articles mentioned the fall in hemoglobin at 24 h. Hence, a larger sample size and additional studies are needed to verify the result. A similar result was observed with regard to the drain volumes (MD = −53.32 mL, 95% CI −116.77, 10.13, *P* = 0.10). This is also reflected in the lesser number of patients requiring blood transfusion (OR = 0.43, 95% CI 0.22, 0.82, *P* = 0.01) in our study. The need for transfusion was an important indicator not only for the blood loss but also for the impact that the surgery had on the general status.

The subgroup analysis showed that the flexion group had significant superiority in terms of ROM at 5–7 days after surgery (MD = 4.97°, 95% CI 1.85, 8.09, *P* = 0.002). However, the follow-up results at 6 weeks showed no significant difference in ROM (MD = −0.29°, 95% CI −3.93, 3.34, *P* = 0.87) between the two groups. The extension group might be associated with increased extravasation of blood within the joint and in the tissues surrounding the knees, which may have lead to increased swelling and poorer recovery.

Many factors contribute to the wound complications, such as blood supply and tension around the wound [27]. Johnson has reported that knee flexion following TKA might increase wound complications by lowering oxygen tension in the wound edges [28]. Eight of the included studies [11, 14, 16, 18–22] mentioned these complications while the remaining two failed to do so [15, 17], and our study did not find any difference in wound complications between the two groups (Table 3).

**Table 3:** Summary of complications reported in ten studies included in the meta-analysis

Included studies	Flexion group	Extension group
Ong [16]	One case of DVT, one case with wound discharge (culture negative)	One case of DVT, one case with wound discharge (culture negative)
Ma [14]	Two cases with superficial wound infection	Two cases with superficial wound infection
Wang [19]	No	No
Madarevic [15]	NA	NA
Li [11]	One case with wound infection	One case with wound infection
Hu [18]	One case with DVT	No
Panni [17]	NA	NA
Zhao [20]	No	No
Antinolfi [21]	One case with wound infection	One case with wound infection
Liu [22]	Three superficial infections, two deep infections	Two superficial infections, two deep infection

*DVT* Deep vein thrombosis, *NA* not available

Compared with a previous systematic review by Faldini [29], there are several improvements in our study. First, this is the first meta-analysis to have quantified the results for the topic. Our methodology is more robust with more stringent inclusion criteria and exclusion of quasi-RCT and non-RCTs from the purview of this meta-analysis for higher validity of the results. Second, six new RCTs comparing these two position after TKA were included in our study. Third, we performed subgroup analyses for fall in hemoglobin and ROM to reduce heterogeneity.

However, there are some limitations in the present meta-analysis. First, the number of studies included in the meta-analysis was relatively small, making it difficult to conduct funnel plots to assess publication bias. Second, there are a lot of variables that could have affected blood loss and ROM after operation, however, a few studies failed to give the details of the confounding factors in their trials. Further studies with more rigorous protocol will be helpful to reduce the bias. Third, the data extracted from the trials had high heterogeneity, with different trials using different angles for knee and hip flexion, which could have affected the results. Finally, most of the included studies failed to give the details about the type of wound dressing used for controlling blood loss, which could have lead to a bias in our findings.

## Conclusions

Our results suggest that flexion of the knee is associated with significantly lesser CBL, lesser HBL, decreased requirement for blood transfusion and better ROM at least in the early postoperative period without increasing in complication rate. However, no significant difference was found in wound drainage at 24 h and ROM at 6 weeks, multicenter randomized controlled trials with large sample size are required to validate our findings.
